# PHYSICAL-MENTAL-ORAL-COGNITIVE HEALTH IN OLDER KOREAN AMERICANS: A MULTISITE STUDY

**DOI:** 10.1093/geroni/igz038.1510

**Published:** 2019-11-08

**Authors:** Yuri Jang, Nan Sook Park, Min-Kyoung Rhee, Hyunwoo Yoon, Yong Ju Cho, miyong T Kim, David A Chiriboga

**Affiliations:** 1 University of Southern California, Los Angeles, California, United States; 2 University of South Florida, Tampa, Florida, United States; 3 Texas State University, san Marcos, Texas, United States; 4 University of Texas at Austin, Austin, Texas, United States

## Abstract

Using data from surveys with older Korean Americans (n = 2,150) conducted at five sites in the U.S. (California, New York, Texas, Hawaii, and Florida), the present study explored the status of physical/mental/oral/cognitive health and its determinants. For each health domain, we examined how self-rating (excellent/very good/good/fair/poor) of health was associated with other domain-relevant indicators (e.g., the number of chronic diseases, symptoms of depression, problems with teeth or gums, or cognitive performance) and sociocultural factors (e.g., socioeconomic status, acculturation, social network, and social cohesion). Geographic variation was also considered. The correlations between self-ratings and domain-relevant indicators in all health domains were significant but moderate. A series of multivariate regression models of self-ratings of physical/mental/oral/cognitive health not only confirmed the effect of the domain-relevant health indicators but also demonstrated a critical contribution of sociocultural determinants. Implications for older immigrants were discussed in terms of place, culture, and context.

